# Development of a method for the detection of zinc in Brassica oleracea using solid phase extraction and size-exclusion chromatography inductively coupled plasma mass spectrometry (SEC-ICP-MS)

**DOI:** 10.1016/j.mex.2021.101428

**Published:** 2021-06-24

**Authors:** Caterina Dell'Aquila

**Affiliations:** Rothamsted Research, West Common, Harpenden, Hertfordshire AL5 2JQ, UK

**Keywords:** SEC-ICP-MS, Vegetables, Extraction, Zinc, Enzyme

## Abstract

The aim of this work is the development of a suitable method to extract and detect zinc-bound compounds from cabbage, broccoli and kale (family Brassicaceae, species oleracea) using solid phase extraction (SPE) and size-exclusion chromatography inductively coupled plasma mass spectrometry (SEC-ICP-MS). Tris [2-Amino-2-(hydroxymethyl)-1,3-propanediol]/hydrochloric acid (Tris/HCl) or ammonium nitrate were used as extractants added to the freeze-dried samples, which were then sonicated and centrifuged. An enzymatic mixture was added to the extracts and then incubated for 5- and 18 h prior to analysis by SEC-ICP-MS. Results showed a good coefficient of variation (CV) of the elution time (0.06-0.9%), concentration (4.7–16.9%) and molecular size (0.4–5.4%). The limit of detection (LOD) and the limit of quantitation (LOQ) were 0.9 μg L−1 and 2.8 μg L−1, respectively. The proposed method is reliable and robust and can be applied to samples with difficult matrices like vegetables and soil.

• Good precision, stability and reproducibility.
• Easy to execute and suitable for analysis of vegetables and other samples with complex matrices, eg. soil.
• This method contributed to good maintenance of the instrument and to minimal cleaning time.

## Graphical abstract

**Figure:**
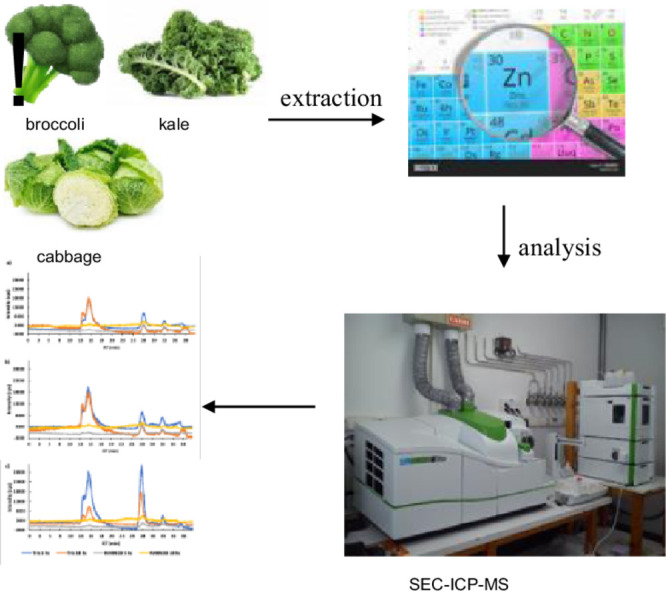

Specifications table

**Table:** 

Subject Area:	Agricultural and Biological Sciences
More specific subject area:	Analytical chemistry, Food science
Method name:	Detection of zinc in vegetables
Name and reference of original method:	Persson, D. P., Hansen, T. H., Laursen, K. H., Schjoerring J. K., Husted, S. (2009). Simultaneous iron, zinc, sulfur and phosphorus speciation analysis of barley grain tissues using SEC-ICP-MS and IP-ICP-MS. Metallomics, 1, 418–426.
Resource availability:	N/A

## Background

Zinc is an essential trace mineral for multiple aspects of metabolism [1] and can play an important role in the immune system and the gene expression [2,3]. Zinc is important for a normal pregnancy and child growth [4], and deficiency is associated with diarrhoea and respiratory infections in children [2]. There is evidence that zinc has an effect on depression and mood, suggesting its supplementation as adjunct to antidepressants therapy for individuals with a diagnosis of major depressive disorder and as a therapy in its own in pre-menopausal women with zinc deficiency [5]. The complex and different matrix of vegetables makes the extraction of Zn-bound compounds difficult even from vegetables within the same species [6]. Different solvents have been used to extract compounds binding zinc from fruit and vegetables. Water, methanol, ethanol, isopropyl alcohol, dimethyl sulfoxide and acetone were used to extract different parts of fruit and vegetables [7,^8^,^9^,^10^,^11^,^12^,13]. However, these extraction methods are complex and require multiple steps. The natural deep eutectic solvents (NADES) is a promising solvent only used to extract minerals from barley [14,15]. Different conditions for the SEC-ICP-MS were used, but not for vegetables [16,17]. In this study two different buffers, Tris/HCl and ammonium nitrate, were used to extract zinc-bound compounds from cabbage, broccoli and kale which are the most consumed vegetables in the UK and then analysed by an adapted method suitable for SEC-ICP-MS.

## Materials and reagents

Ammonium nitrate, (Sigma-Aldrich, Poole, Dorset, UK)

Tris [2-Amino-2-(hydroxymethyl)-1,3-propanediol], (Sigma-Aldrich, Poole, Dorset, UK)

Phytase from wheat, (Sigma-Aldrich, Poole, Dorset, UK)

Protease, (Type XIV from Streptomyces griseus), (Sigma-Aldrich, Poole, Dorset, UK)

Xylanase, (Thermomyces lanuginosus), (Sigma-Aldrich, Poole, Dorset, UK)

Cellulase, (Trichoderma reesei), (Sigma-Aldrich, Poole, Dorset, UK)

Tri-glycine, (Sigma-Aldrich, Poole, Dorset, UK)

Vitamin B12, (Sigma-Aldrich, Poole, Dorset, UK)

Cytochrome, (Sigma-Aldrich, Poole, Dorset, UK)

Apoferritin, (Sigma-Aldrich, Poole, Dorset, UK)

Blue dextran, (Sigma-Aldrich, Poole, Dorset, UK)

Freeze-dried savoy cabbage (Brassica oleracea var. sabauda), freeze-dried broccoli (Brassica oleracea var. italica) and freeze-dried curly kale (Brassica oleracea var. acephala), (Norwich Medical School, University of East Anglia, Norwich Research Park, Norwich, UK)

Elemental solution (Zn), Fisher Scientific (Loughborough, Leicestershire, UK)

## Method details

### 1. Sample preparation

Savoy cabbage (Brassica oleracea var. sabauda), broccoli (Brassica oleracea var. italica) and curly kale (Brassica oleracea var. acephala) were purchased from different supermarkets. About 400 g of savoy cabbage (chopped in wedges) and broccoli (chopped in heads) were boiled in 1.2 L Milli-Q (18.2 MQ) water for about 15 min, while 360 g of curly kale were boiled in about 1.6 L Milli-Q (18.2 MQ) water for 8 min. After cooking, the vegetables were drained for 15 min at room temperature, placed in bags, frozen at minus 20 °C, freeze-dried (Coolsafe 110-4 pro, ScanVac), and finely ground. The ground samples were stored, at the Norwich Medical School, in the dark in sealed bags at 4 °C in a desiccator, with silica gel beads to prevent moisture.

Two batches of samples (100 mg) were extracted twice by solid phase extraction with 2 mL of buffer (200 mM ammonium nitrate pH 7.6 or 50 mM Tris/HCl pH 7.5) in plastic tubes, sonicated at 20% power for 2 min. The extracts within the same batch and from the same vegetable were combined and centrifuged at 4000 g/min at 4 °C for 10 min.

### 2. Enzymatic digestion

The enzymatic mixture (3 mL) (Table 1) was prepared in a plastic tube and added to 2 mL of the extract to have a final volume of 5 mL. The samples were incubated for 5 h at 37 ᴼC with gentle shaking (120 rpm) in an orbital incubator. After 5 h, 1 mL of the incubated samples was taken and kept in a refrigerator for SEC-ICP-MS. The rest of the incubated samples was put back in the incubator for another 13 h to have a total of 18 h. After 13 h, all the incubated samples were centrifuged at 4000 g/min for 10 min and analyzed by SEC-ICP-MS. All samples were diluted 1:10 prior to analysis.

**Table 1:** Enzymatic mixture used for the digestion.

Enzyme	Origin	Activity (U/g)
Phytase (30 U/g)	wheat	20
Protease (3500 U/g)	Streptomyces griseus	175
Xylanase (2500 U/g)	Thermomyces lanuginosus	280
Cellulase (700 U/g d=1.2 g/mL)	Trichoderma reesei	440

### 3. Analytical instrumentation

Separation of samples was performed via size-exclusion chromatography [20] using a high-performance liquid chromatography (HPLC) (PerkinElmer LC 200 Series HS, Seer Green, Bucks, UK), a Flexar ultraviolet/visible (UV/VIS) detector operating at 280 nm and a high-pressure peristaltic pump equipped of PEEK tubing (0.17 mm id) and operating at a flow rate of 0.6 mL min^−1^. Detection and quantification of elements were made using Chromera software (PerkinElmer v. 4.1.0). The column was a Superdex Peptide 10/300 GL (10Å~300 mm, GE Healthcare Bio-Sciences, Uppsala, Sweden) with an optimum separation range between 100 and 7000 Da. Samples were analyzed using an ICP-MS (PerkinElmer NexION300XX, Seer Green, Bucks, UK) equipped with a glass Meinhard nebulizer, a quadrupole mass spectrometer and a collision cell. The SEC-ICP-MS settings were as in Table 2. Ammonium nitrate (200 mM, pH 7.6), as mobile phase, was prepared daily by dissolving 16 g of ammonium nitrate in 1 L of Purite ultrapure water and the pH was adjusted with ammonium hydroxide before degassing by vacuum filtration using 0.2 μm filters (Millipore, Watford, UK). Samples (100 μL) were injected into the column at room temperature and the analysis was performed in isocratic mode. After five runs a series of five repetitive 20 µL aliquots of water was injected over 20 min, without delay between injections, followed by one 100 µL aliquot of ammonium nitrate (200 mM at pH 7.6) to re-equilibrate the column. The same series of five repetitive 20 µL aliquots of water was injected, at the end of the batch, using water as mobile phase. An average of seven replicates per sample was carried out.

**Table 2:** Instrumentation settings.

ICP-MS	

Gas flow	1 L/min
Auxiliary gas flow	1.2 L/min
Plasma flow	18 L/min
RF power	1600 Watts
Cell gas flow (He)	3.9 mL/min
Mode	Collision cell
HPLC	

Column	Superdex Peptide 10/300 GL
Eluent	NH4NO3 (200 Mm, pH 7.6)
Flow rate	0.6 mL/min
Mode	isocratic
Sample volume	100 µL
Wavelength	280 nm

### 4. Quality control

**Fig. 1:**
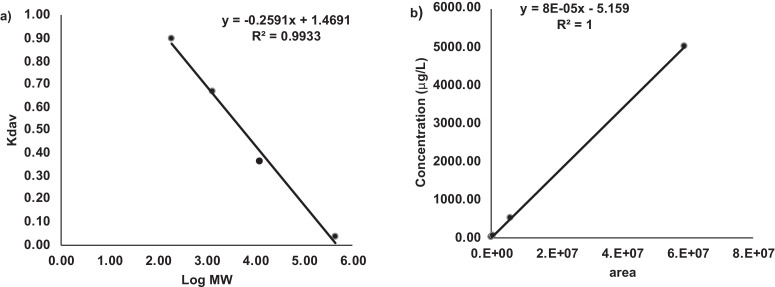
Calibration curve for a) molecular mass and b) quantification.

Zinc is a common contaminant which can be problematic for trace analysis. In this study, plastic bottles were used for the solvents and metal-free glass vials were used for the samples to avoid metal contamination which was checked by injecting a blank made of 2% HNO3. External calibration for quantification provided a linear range with an excellent correlation coefficient (R = 1) and was performed by injecting six concentrations of zinc standard solution (0, 0.050, 0.5, 5, 10 and 100 mg L^−1^), dissolved in 2% HNO3, into the ICP-MS via HPLC but without the column, which was detected as Zn^64^ isotope. The standards for mass calibration were triglycine (0.189 kDa), vitamin B12 (1.35 kDa), cytochrome (12.4 kDa), apoferritin (443 kDa) and blue dextran (2000 kDa) dissolved in an appropriate amount of mobile phase and filtered. A log-linear regression curve, with a correlation coefficient R = 0.99, was obtained by plotting the logarithm of the molecular size versus the coefficient of distribution (K_d_) (Fig 1). Data are presented as the average of seven intra- and inter-day analyses. The limit of detection (0.9 μg L^−1^) and the limit of quantitation (2.8 μg L^−1^) were determined using LOD=3.3*(SD/m) and LOQ=10*(SD/m) where SD is the standard deviation of the intercept and m is the slope of the calibration curve. Reproducibility and precision were measured by the coefficient of variation (CV) of the retention time of the smallest peaks eluted at about 28 and 32 min (Table 3). Determination of the accuracy is difficult because it is impossible to obtain a sample with a known amount of zinc. For other kind of matrices, like plasma for example, the accuracy can be determined by spiking a zinc-free plasma with known concentrations; for vegetables this is not possible because most of the vegetables contain a certain amount of zinc and therefore zinc-free samples are not available. It is impossible to obtain a sample with a known amount of zinc because of the large variation of its amount in vegetables which depends on different factors, e.g., environment, soil, geographic area and so on.

**Table 3:** Coefficient of variation of the retention time, concentration and molecular size of the peaks eluted after 28 and 32 min.

	CV (%)					
	28 min			32 min		
	RT	concentration	MW	RT	concentration	MW
Cabbage	0.12	5.5	0.4	0.12	8.0	0.4
Broccoli	0.06	4.7	0.8	0.25	11.1	1.0
Kale	0.17	8.9	3.3	0.92	16.9	5.4

## Extraction

**Fig. 2:**
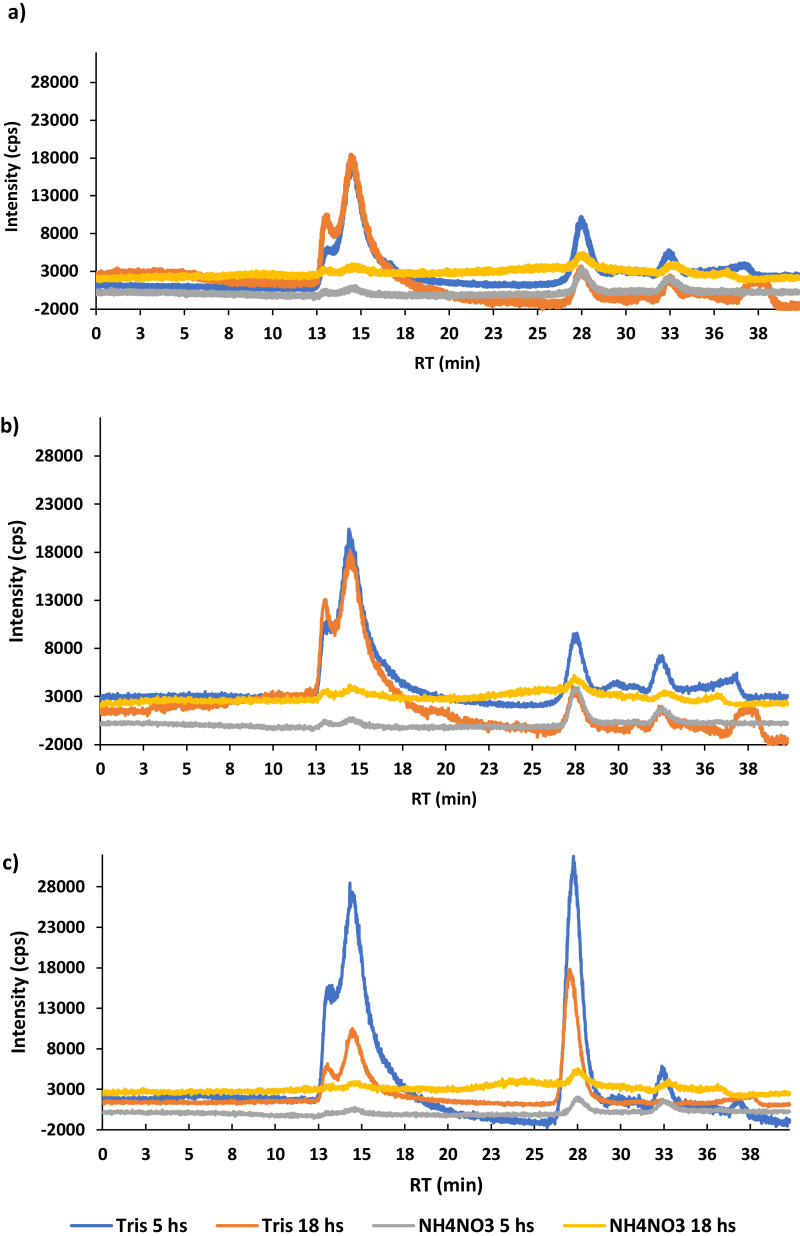
Comparison between the samples extracted with Tris/HCl and NH4NO3 and digested for 5 h and 18 h. SEC-ICP-MS: mobile phase ammonium nitrate (200 mM, pH 7.6), flow rate of 0.6 mL min^−1^, l = 280 nm. a) cabbage, b) broccoli, c) kale.).

**Fig. 3:**
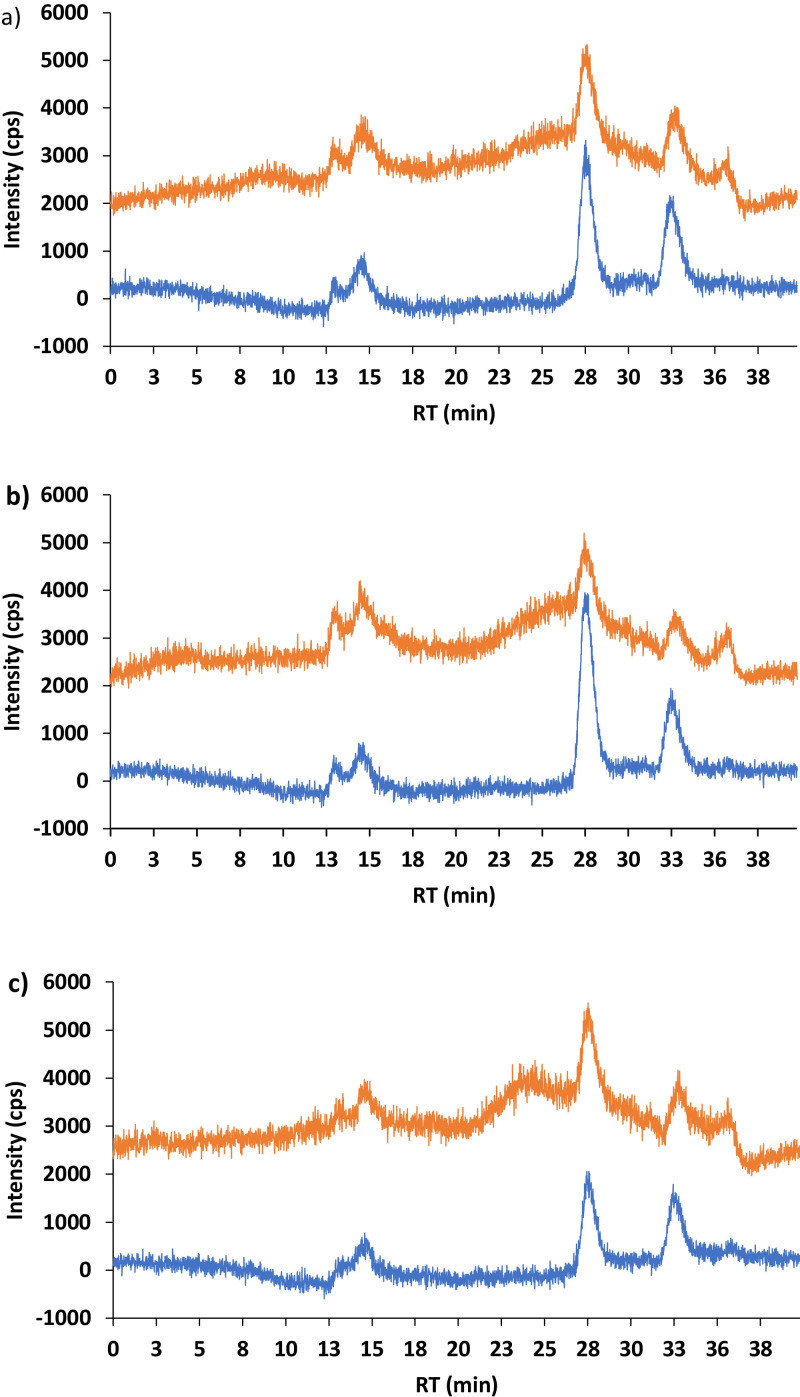
Comparison between 5 h (blue line) and 18 h (orange line) digestion of (a) cabbage, (b) broccoli and (c) kale with NH4NO3 (low intensity chromatograms in Fig.2). SEC-ICP-MS: mobile phase ammonium nitrate (200 mM, pH 7.6), flow rate of 0.6 Ml. (For interpretation of the references to color in this figure legend, the reader is referred to the web version of this article.).

Tris/HCl buffer works better for the extraction of species containing zinc (Fig. 2) than ammonium nitrate which gave a high noise background threshold (Fig. 3). Tris/HCl is a common buffer used to extract metal-bound species from different genotypes of wheat [18,24], probably for its lysis properties which can explain its high extraction ability. Ammonium nitrate is used as a fertilizer in agriculture to provide a source of nitrogen which stimulates the plant growth, and it was chosen because it was considered a suitable solvent to extract water-soluble plant available trace elements from soil [21,22] and toxic elements from contaminated grassland soils to assess metal mobility in soil [19]. It was expected ammonium nitrate to extract Zn-bound species better than Tris/HCl, because the ammonia [23] formed by its dissociation leads to the formation of soluble metal ammine complexes, mainly occurring in the soil, which are available for plants [19]. On the contrary, in this study ammonium nitrate showed a very low level of extraction compared to Tris/HCl buffer.

## SEC-ICP-MS

Ammonium nitrate is not a good extractant, but it is excellent as mobile phase for its stability, reproducibility and precision. The challenge of this study was to find a method suitable for the ICP and the HPLC instruments at the same time. Ammonium nitrate has a good buffer capacity at a pH range of 7.6–7.8, it is very soluble in water and does not precipitate in the column or in the ICP system. The five repetitive 20 µL aliquots injections are a push-through way to rinse the column, and the tubing, chamber and injector of the ICP. Precipitate and dirt accumulation on the cone, injector, nebulizer and torch of the ICP are common along with blockage of the tubing. This method is reliable for samples with difficult and complex matrices and showed consistency of the results not only in vegetables but also in soil.
